# Myofibroblast Differentiation and Enhanced Tgf-B Signaling in Cystic Fibrosis Lung Disease

**DOI:** 10.1371/journal.pone.0070196

**Published:** 2013-08-12

**Authors:** William T. Harris, David R. Kelly, Yong Zhou, Dezhi Wang, Mark Macewen, James S. Hagood, J. P. Clancy, Namasivayam Ambalavanan, Eric J. Sorscher

**Affiliations:** 1 Department of Pediatrics, University of Alabama at Birmingham, Birmingham, Alabama, United States of America; 2 Department of Pathology, University of Alabama at Birmingham, Birmingham, Alabama, United States of America; 3 Department of Medicine, University of Alabama at Birmingham, Birmingham, Alabama, United States of America; 4 Center for Metabolic Bone Disease Histomorphometry and Molecular Analyses Core Laboratory, University of Alabama at Birmingham, Birmingham, Alabama, United States of America; 5 Department of Pediatrics, University of California at San Diego and Rady Children's Hospital of San Diego, San Diego, California, United States of America; 6 Department of Pediatrics, Cincinnati Children's Hospital Medical Center, Cincinnati, Ohio, United States of America; University of Giessen Lung Center, Germany

## Abstract

**Rationale:**

TGF-β, a mediator of pulmonary fibrosis, is a genetic modifier of CF respiratory deterioration. The mechanistic relationship between TGF-β signaling and CF lung disease has not been determined.

**Objective:**

To investigate myofibroblast differentiation in CF lung tissue as a novel pathway by which TGF-β signaling may contribute to pulmonary decline, airway remodeling and tissue fibrosis.

**Methods:**

Lung samples from CF and non-CF subjects were analyzed morphometrically for total TGF-β_1_, TGF-β signaling (Smad2 phosphorylation), myofibroblast differentiation (α-smooth muscle actin), and collagen deposition (Masson trichrome stain).

**Results:**

TGF-β signaling and fibrosis are markedly increased in CF (p<0.01), and the presence of myofibroblasts is four-fold higher in CF vs. normal lung tissue (p<0.005). In lung tissue with prominent TGF-β signaling, both myofibroblast differentiation and tissue fibrosis are significantly augmented (p<0.005).

**Conclusions:**

These studies establish for the first time that a pathogenic mechanism described previously in pulmonary fibrosis is also prominent in cystic fibrosis lung disease. The presence of TGF-β dependent signaling in areas of prominent myofibroblast proliferation and fibrosis in CF suggests that strategies under development for other pro-fibrotic lung conditions may also be evaluated for use in CF.

## Introduction

Cystic fibrosis (CF) is a common inherited disease among Caucasians, affecting approximately 1 in 3000 births [1]. CF affects 30,000 children and young adults in the United States [2]. The progression of CF lung disease is variable, even among individuals sharing the same mutation in the cystic fibrosis transmembrane regulator (CFTR) [3]. Recent progress in animal models such as the cystic fibrosis pig have expanded the understanding of CF pathogenesis beyond mucus obstruction and infection/inflammation to include defects in hormone-mediated growth, development, and airway remodeling [4]–[6].

CF patients with specific polymorphisms in TGF-β_1_ (a potent pro-fibrotic cytokine) have a significantly increased odds ratio of severe lung disease [7]. Multi-organ fibrosis (liver cirrhosis, pancreatic fibrotic obliteration, vas deferens obstruction) is well described in CF (the formal name of the disease is “cystic fibrosis of the pancreas”) [8]. TGF-β is a known mediator of fibroblast pathobiology in human lungs [9], and is also a modifier of disease severity among CF individuals [7], [10]–[12]. However, TGF-β signaling and the mechanisms underlying development of lung fibrosis in CF have not been characterized previously.

The myofibroblast (a fibroblast with α-smooth muscle actin among other contractile elements) has been identified as a key mediator of idiopathic pulmonary fibrosis (IPF) and other profibrotic conditions [13]–[15]. This distinct myofibroblast phenotype is TGF-β dependent and arises secondary to chronic epithelial injury or inflammation, two well described features of CF respiratory deterioration. In this study, we demonstrate intense TGF-β signaling and provide the first quantitative description of the myofibroblast phenotype in CF lungs. These results point to a novel explanation for TGF-β as a genetic modifier of CF lung disease and indicate emerging anti-fibrotic therapies under development for disease such as systemic sclerosis and idiopathic pulmonary fibrosis [16] should also be considered as interventions to diminish tissue scarring and respiratory compromise in CF.

## Methods

### Institutional approval

Use of human tissue was approved by the University of Alabama at Birmingham (UAB) Institutional Review Board (IRB protocol # X081204008) prior to conducting these studies. De-identified tissue specimens were obtained through the UAB Airway Tissue Procurement core facility (CF, IPF, normal) and the National Disease Research Interchange (ndriresource.org). The use of these de-identified tissue specimens was reviewed by the UAB IRB and deemed Not Human Subjects Research.

### Tissue Procurement

CF patients scheduled for lung transplantation provided informed written consent to the UAB Airway Tissue Procurement core facility for use of their pulmonary tissue in research studies. De-identified lung specimens from non-CF individuals were obtained from both the UAB (autopsy and failed donor tissue) and the National Disease Research Interchange (ndriresource.org), Pro-fibrotic control samples were obtained from subjects with idiopathic pulmonary fibrosis (IPF) who provided written informed consent to the UAB Airway Tissue Procurement core facility at the time of lung transplantation.

### Immunohistochemistry

Formalin-fixed, paraffin-embedded blocks were sectioned at 4 µm. Sections of parenchyma were taken from the periphery of each lobe to control for regional heterogeneity. Heat-induced epitope retrieval with 0.02 M of citrate buffer (pH 6.0) at 97°C for 20 minutes was performed and immunohistochemistry accomplished with a semi-automated ThermoScientific (Fremont CA) immunostainer (Autostainer 720) and polymer system (UltraVision LP). Antibodies to TGF-β_1_ (SC-146 1∶100 dilution, Santa Cruz Biotechnology), phosphorylated Smad2 (#3101 Ser465/467 1∶100 dilution, CellSignaling), and α-smooth muscle actin (AB-1 1∶100, Biocarta) were used for detection by diaminobenzine tetrachloride (DAB). Tissues were counterstained with hematoxylin, and negative control slides (lacking primary antibody) prepared in all cases.

### Morphometry

Immunohistochemical (IHC) findings were assessed using both semi-quantitative and quantitative measures. Semi-quantitative analysis defined a 1–5 scale: 1+ (minimal staining), 2+ (moderate staining), 3+ (prominent), 4+ (extensive), 5+ (ubiquitous). Quantitative analysis for TGF-β, Masson trichrome and α-SMA staining utilized MetaMorph software (Molecular Devices, LLC, Sunnyvale, CA) with automated thresholding for a positive stain in the region of interest as described previously [17]. For each tissue slide, 10 random fields (100×) were analyzed in a blinded fashion. Quantitative histomorphometry for pSmad2 nuclear staining utilized Bioquant Image Analysis software (RTM, Nashville, TN) from 10 random fields obtained at 200× as previously described [18]. In brief, the software identifies positive stained cells with an automated threshold tool confirmed by reader verification and then quantifies the extent of positive staining in each region of interest. Measurement techniques were reviewed and validated by the UAB Histomorphometry and Molecular Analysis Core.

### Statistics

Data distribution was assessed using the D'Agostino-Pearson normality test. Parametric data was analyzed with a t-test for comparison of two variables and ANOVA with Tukey-Kramer post-test analysis for multiple comparisons. Non-parametric data was evaluated by Mann-Whitney for comparison of two variables and Kruskal-Wallis with Dunn's post-test analysis for multiple comparisons. For all analytical studies, significance was assigned to p≤0.05.

## Results

### Demographics

Human lung tissue was procured from 6 CF patients, 26 non-CF individuals, and 8 patients with idiopathic pulmonary fibrosis (IPF). Approximately 5 sections per CF patient were reviewed to account for regional heterogeneity. The 26 non-CF tissue specimens (human normal lung, HNL) were obtained from the National Disease Research Interchange (NDRI, n = 16), UAB autopsy specimens (n = 5), and UAB failed lung transplant donors (n = 5). Overall patient characteristics are described in Table 1.

**Table 1:** Demographics.

Tissue Diagnosis	N	Age	Gender
	(subjects)	(years)	(% male)
CF	6	28.3±3.6	67%
HNL (total)	26	48.5±4.4	54%
NDRI	16	52.8±6.0	63%
UAB autopsy	5	52.2±5.9	40%
UAB Failed Donor	5	35.9±9.3	40%
IPF	8	58.8±2.5	75%

CF: cystic fibrosis; HNL: Human normal lung tissue; NDRI: National Disease Research Interchange; a national organization specializing in tissue procurement for research purposes; UAB: University of Alabama at Birmingham; IPF: idiopathic pulmonary fibrosis.

### TGF-β_1_ expression

Both CF and HNL tissue stained prominently for total (latent and active) TGF-β_1_ (data not shown). Bronchial epithelial cells, lymphoid inflammatory cells and luminal secretions were strongly positive. Extracellular matrix also stained for the cytokine. No significant differences in tissue staining for total TGF-β_1_ were detected between CF versus HNL tissue either by semi-quantitative or quantitative analysis.

### TGF-β signaling (pSmad2)

**Figure 1:**
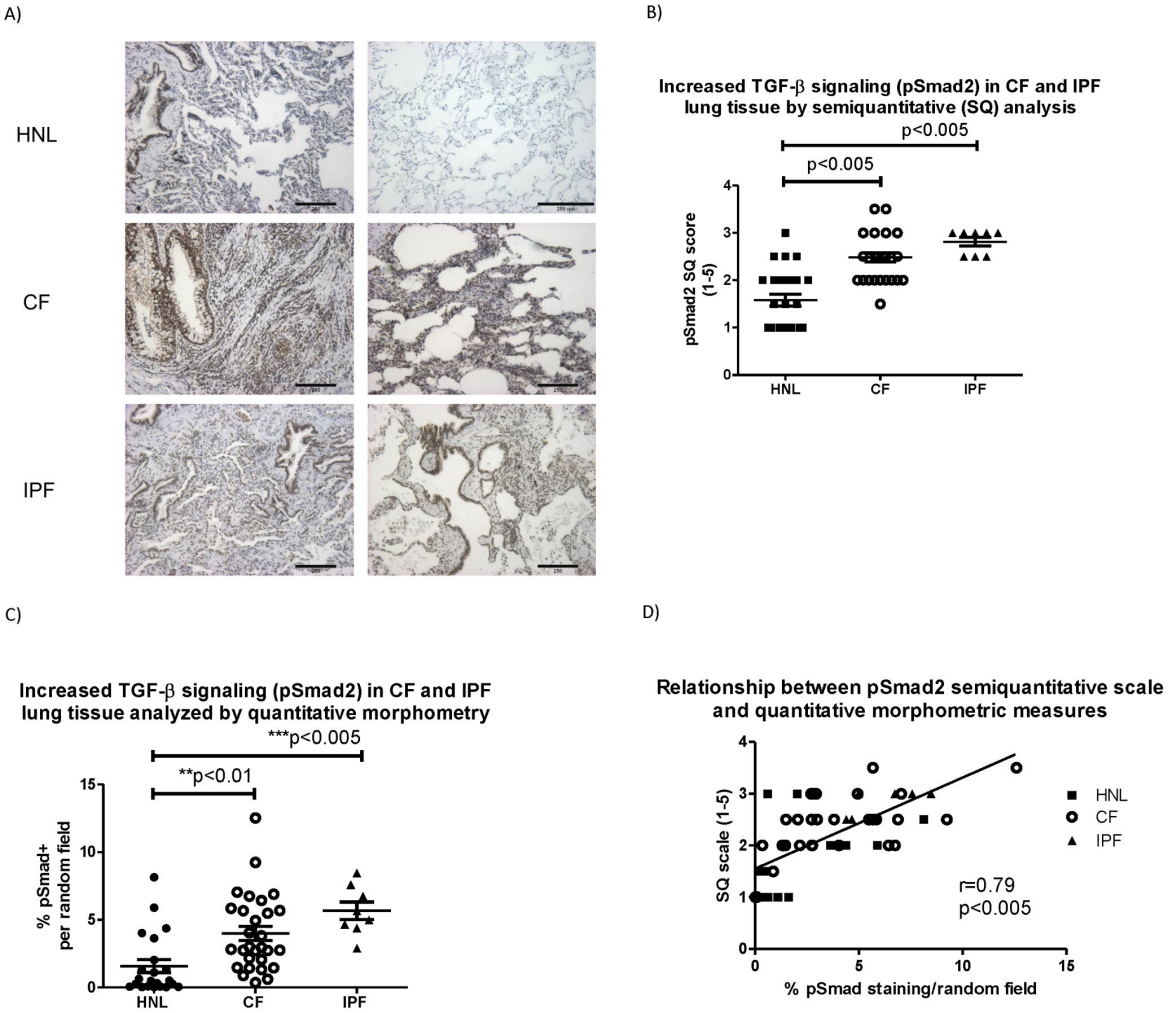
Increased TGF-β signaling in lung tissue from cystic fibrosis (CF) and idiopathic pulmonary fibrosis (IPF) compared to non-CF human normal lung tissue (HNL). A) Immunohistochemical staining for phosphorylated Smad2 (pSmad2 shown in brown). The downstream signal of TGF-β is significantly increased in CF compared to HNL tissue and comparable to that seen in IPF. Images are shown at approximately 100× from 2 separate CF, HNL and IPF specimens (bar = 250 µm). B) Comparison of TGF-β signaling (pSmad2 staining) by semiquantitative score (1–5). C) Comparison of TGF-β signaling (pSmad2 expression) by quantitative morphometry. D) Correlation between semiquantitative scale (1–5 scale) and morphometric measures of pSmad2.

Staining for pSmad2 (a marker for signaling by activated TGF-β) was significantly increased in CF versus HNL by semi-quantitative scoring (CF: 2.5±0.1, HNL: 1.6±0.1, p<0.005) and comparable to that seen for the classical pulmonary fibrotic disease, IPF (2.8±0.1) (Figure 1A and 1B). Quantitative morphometry conducted in a blinded fashion for pSmad2 in each of ten random fields at 200× confirmed increased pSmad2 in CF vs. HNL tissue (CF: 4.0%, HNL: 1.5%, p<0.01) (Figure 1C) with significant correlation between the semiquantitative and quantitative measures (r = 0.79, p<0.005) (Figure 1D).

### Fibrosis in CF lung parenchyma

**Figure 2:**
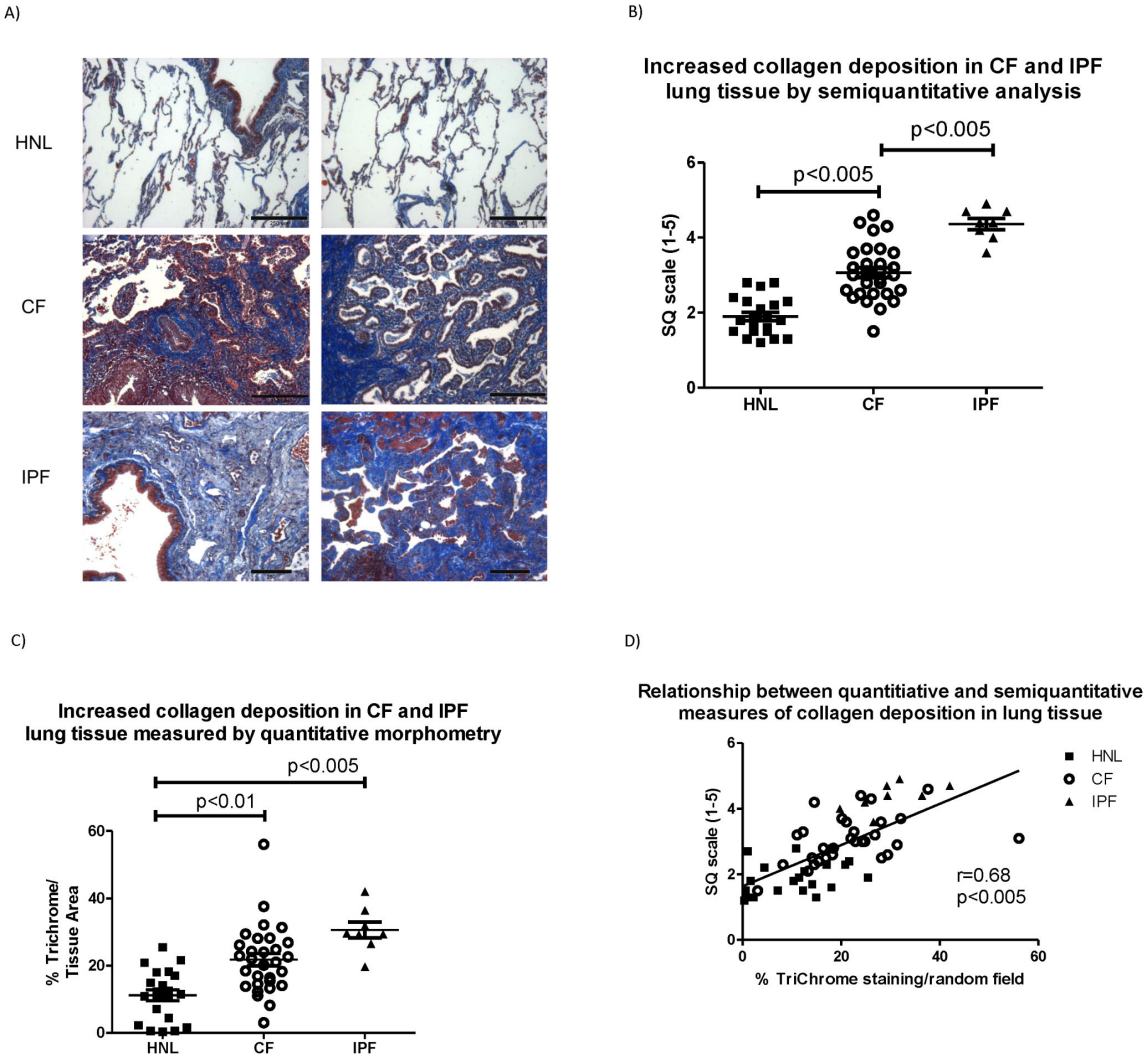
Increased peripheral lung fibrosis in cystic fibrosis (CF). A) Histochemical staining for fibrosis (Masson trichrome stain, collagen shown in blue) in human normal lung (HNL), cystic fibrosis (CF) and idiopathic pulmonary fibrosis (IPF). Images are shown at approximately 100× from 2 separate HNL, CF and IPF specimens (bar = 250 µm). B) Comparison of collagen deposition (Masson trichrome stain) by semiquantitative analysis and C) quantitative morphometry. D) Correlation between quantitative morphometry (% collagen deposition) and semiquantitative analysis (1–5 scale).

A surprisingly small number of previous studies have quantified the extent of fibrosis in CF pulmonary tissue. Figure 2 demonstrates that fibrosis is markedly increased in CF (compared to HNL) lung specimens, extending beyond the peribronchiolar interstitium and obliterating the surrounding septal parenchyma. By the semi-quantitative metric, CF lung tissue had significantly more fibrosis than HNL (CF: 3.1±0.1; HNL 1.9±0.1, p<0.005) (Figure 2B). Quantitative analysis by tissue morphometry led to the same conclusion, with a greater percentage of fibrosis present in each random field at 100× (CF: 21.8±1.8%, HNL: 11.2±1.6%, p<0.01) (Figure 2C). Tissue morphometry and semi-quantitative analysis were again well-correlated (r = 0.68, p<0.005) (Figure 2D).

### Myofibroblast differentiation in human CF lung tissue

**Figure 3:**
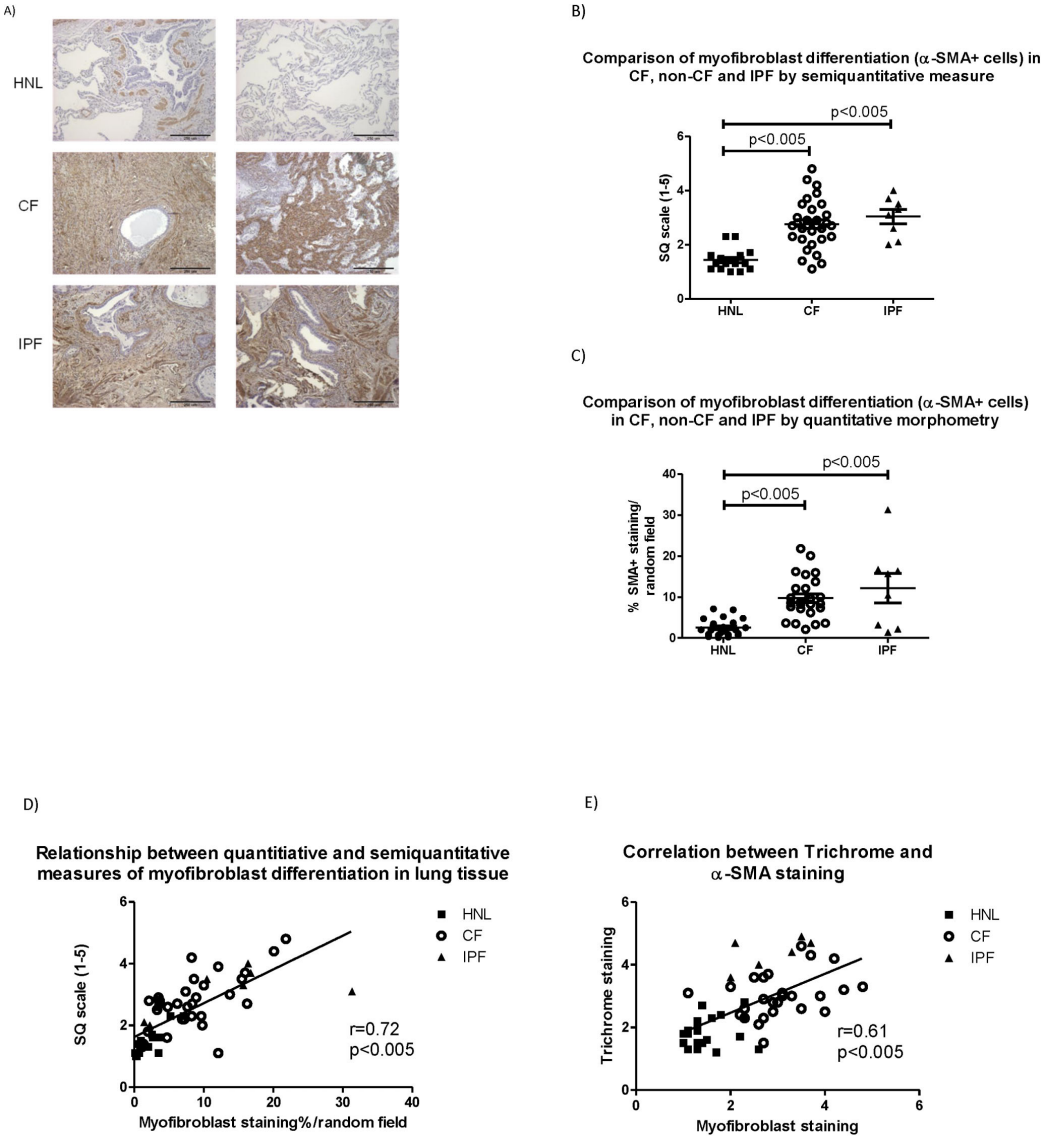
Myofibroblast differentiation in human normal (HNL), cystic fibrosis (CF) and idiopathic pulmonary fibrosis (IPF) lung tissue. A) Immunohistochemical staining for α-smooth muscle actin (α-SMA), the contractile element in myofibrobroblasts. Myofibroblast accumulation is significantly accentuated in cystic fibrosis (CF) and idiopathic pulmonary fibrosis (IPF) compared to non-diseased controls. Images at 100× (bar = 250 µm). B) Comparison of myofibroblast differentiation in HNL, CF and IPF lung tissue analyzed semi-quantitatively. C) Comparison of myofibroblast differentiation in HNL, CF and IPF lung tissue analyzed by quantitative morphology. D) Correlation between semiquantitative and quantitative measures of myofibroblast differentiation in HNL, CF and IPF lung tissue specimens. E) Myofibroblast differentiation versus tissue fibrosis in HNL, CF and IPF lung tissue specimens.

Myofibroblasts were increased in human CF lung tissue compared to HNL, as determined by IHC for α-SMA (Figure 3). In blinded semiquantitative analysis, CF lung tissue demonstrated myofibroblast prominence (CF: 2.8±0.2, HNL 1.4±0.1, p<0.005) (Figure 3B). Quantitative analysis indicated myofibroblasts occupied a higher percentage of each random field (100×) in CF vs. non-diseased individuals (CF: 9.8±1.0%; HNL 2.5±0.4%, p<0.005) (Figure 3C). Morphometry correlated with semi-quantitative analyses (r = 0.72, p<0.005) (Figure 3D). The magnitude of myofibroblast proliferation also tracked with the extent of lung fibrosis across lung tissue specimens (r = 0.61, p<0.005) (Figure 3E).

### Comparison between CF and idiopathic pulmonary fibrosis (IPF)

**Figure 4:**
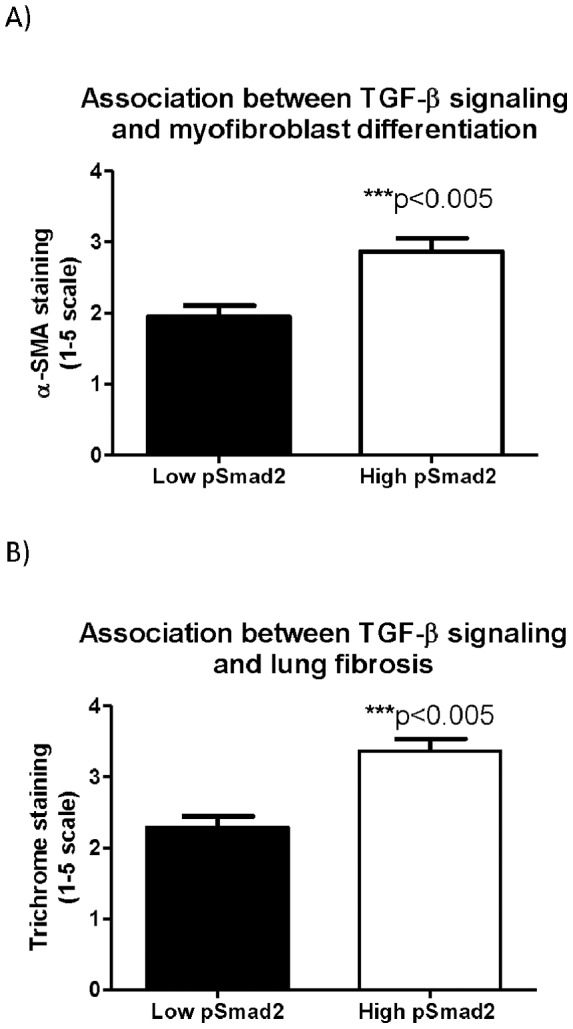
Association between TGF-β signaling, myofibroblast differentiation and tissue fibrosis. A) Myofibroblast differentiation is significantly increased in lung tissue with increased TGF-β signaling (defined as ≥ positive control on semiquantitative scale) B) Lung fibrosis is similarly increased in lung tissue with increased TGF-β signaling.

Myofibroblast differentiation is an established contributor to fibrosis in idiopathic pulmonary fibrosis [14], [19], a disease in which profound respiratory failure is attributed to a seminal defect in parenchymal fibrosis. Increased TGF-β signaling is known to elicit myofibroblast differentiation among patients with IPF [20]. We therefore compared the extent of parenchymal fibrosis, myofibroblast differentiation and TGF-β signaling in CF versus IPF lung specimens. In both the CF and IPF lung, TGF-β signaling (pSmad2 staining) was increased compared to normal lung controls (Figure 1) with a non-significant trend toward increased TGF-β signaling in IPF vs. CF (p = 0.07). In lung samples with significant TGF-β signaling (semiquantitative score≥2.5), both myofibroblast differentiation and tissue fibrosis were significantly increased (p<0.005) (Figure 4).

Tissue fibrosis in CF was below levels observed in IPF (Figure 2), but the extent of myofibroblast differentiation in CF was comparable to IPF (Figure 3), a condition in which TGF-β dependent myofibroblast differentiation is considered etiologic [20]. Notably, a subset of CF specimens exhibited TGF-β signaling, tissue fibrosis and myofibroblast differentiation similar to that seen in IPF (Figures 1, 2, and 3). Figure 3E demonstrates the significant correlation between tissue fibrosis and myofibroblast differentiation across all (n = 40) lung specimens.

## Discussion

In this study, we demonstrate that TGF-β signaling and myofibroblast differentiation are increased in the CF lung and approach pathogenic levels observed in patients with idiopathic pulmonary fibrosis. In IPF, it is well established that excessive TGF-β signaling and myofibroblast differentiation serve as the proximate cause of respiratory failure [20]–[22]. The present experiments were therefore designed to apply emerging mechanistic knowledge regarding lung fibrosis and test the relevance of the TGF-β profibrotic pathway to cystic fibrosis pulmonary disease. In CF lungs, TGF-β signaling was markedly increased as judged by pSmad2 expression, increased myofibroblast differentiation and tissue fibrosis. These findings point to aberrant myofibroblast differentiation as an important contributor to airway remodeling in late-stage CF, and suggest the myofibroblast as a novel mediator of CF pulmonary destruction. The results also provide important insight regarding the process of tissue scarring and remodeling that contributes to CF respiratory decline.

Myofibroblast differentiation occurs normally in the setting of tissue injury, TGF-β stimulation, and mechanical strain. The myofibroblast contributes to healing by approximating wound edges and promoting extracellular matrix formation. In health, myofibroblasts undergo apoptosis following resolution of tissue injury. In diseases such as IPF, aberrant myofibroblast persistence results in tissue fibrosis and parenchymal contracture. Recently, Ulrich et al [23] noted increased myofibroblasts and ceramide deposition in peripheral CF alveolar tissue. Ziobro et al [24] subsequently demonstrated a palliative effect of blunting ceramide accumulation in the CF murine model, but the functional significance of myofibroblasts in CF lung pathophysisology has not been further developed. The present experiments indicate the importance of TGF-β mediated pulmonary fibrosis in human cystic fibrosis lung disease.

We have previously demonstrated elevated protein levels of TGF-β in bronchoalveolar lavage fluid (BALF) in CF vs. non-CF pediatric patients [25]. In CF, we showed that TGF-β in lavage fluid is associated with increased airway inflammation, recurrent hospitalizations, and diminished lung function [25]. We also found that increased plasma TGF-β in CF could be used to predict impaired lung mechanics, and that diminished levels of TGF-β were observed following clinical responsiveness to parenteral antibiotic therapy [26]. TGF-β has multiple biologic functions linked to airway remodeling, wound healing and fibrosis, including enhanced cellular migration, immunomodulation and extracellular matrix synthesis [27]. To mediate biologic effect, latent TGF-β must be activated to bind the TGF-β receptor complex which transmits a signal chiefly through Smad protein phosphorylation (pSmad2/pSmad3) [27]. Our findings establish TGF-β activation is markedly upregulated in CF lung disease, and provide new insight regarding the well-described but not well-understood role of TGF-β as a genetic modifier of cystic fibrosis pulmonary decline.

**Figure 5:**
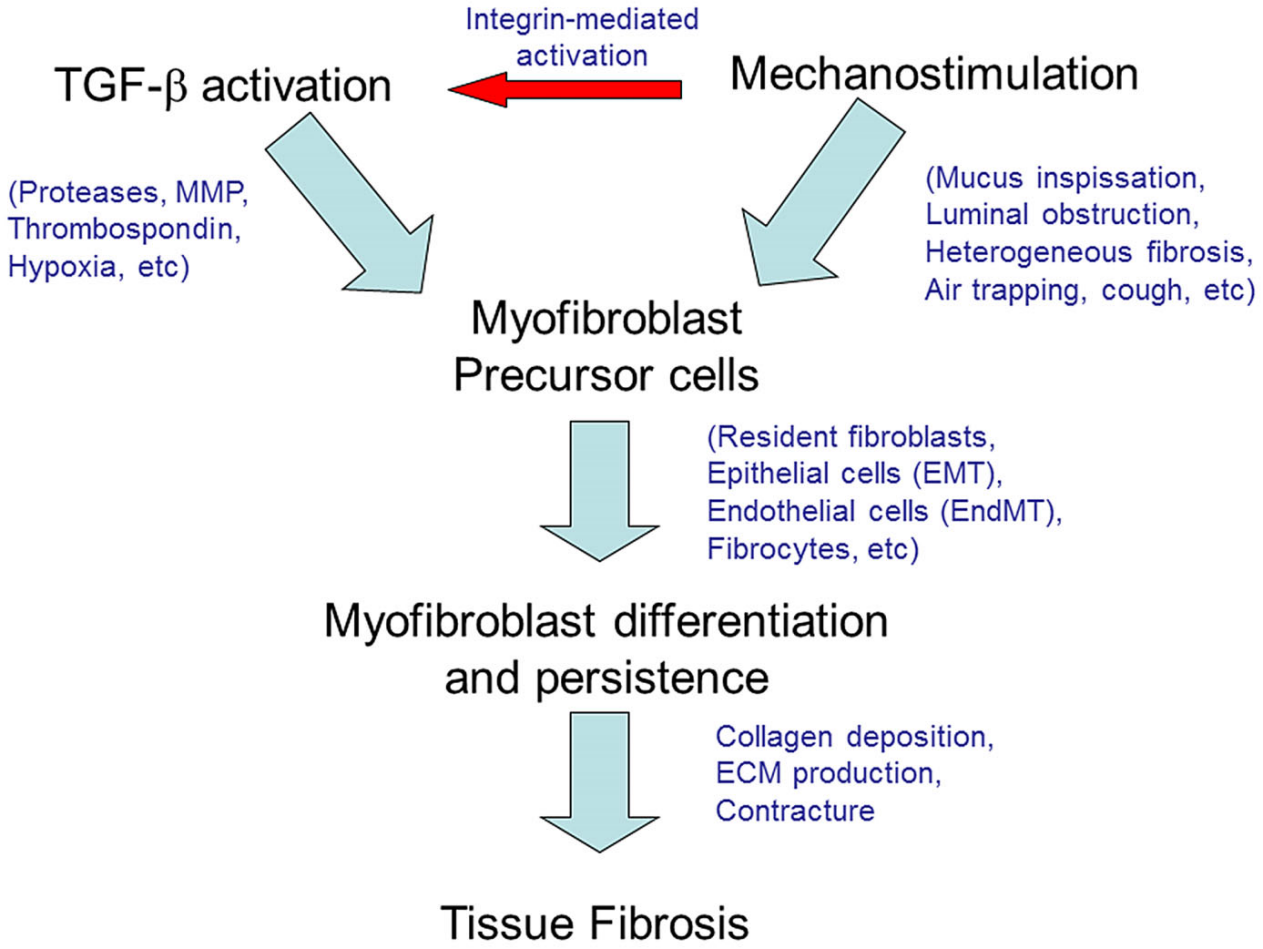
Schematic model depicting myofibroblast differentiation in cystic fibrosis (CF). TGF-β signaling and mechanostimulation in CF lungs induce precursor cells such as resident fibroblasts to undergo myofibroblast differentiation. TGF-β activation is robust and likely multifactorial due to well-established mechanisms including integrin expression and proteases such as the matrix metalloproteases (MMPs). Mechanical strain (e.g. from luminal obstruction, tissue fibrosis and persistent coughing) further augments TGF-β activation and contributes to development and persistence of the myofibroblast phenotype. Sources of myofibroblast precursors include resident lung tissue fibroblasts, circulating fibrocytes, epithelial mesenchymal transition (EMT) or endothelial mesenchymal transition (EndMT). Persistence of the myofibroblast leads to progressive tissue fibrosis with collagen production, extracellular matrix synthesis and tissue contracture.

A model depicting the current findings and their pathologic significance is shown in Figure 5. From a mechanistic standpoint, myofibroblast differentiation is induced in the setting of increased TGF-β signaling and mechanical strain, both of which are prominent in CF lungs. Well-described factors that increase TGF-β signal transduction in cystic fibrosis airways include local hypoxia [28], [29], persistent epithelial injury, and increased protease activity [30]–[32]. Furthermore, myofibroblasts themselves promote TGF-β activation via contraction [33], and mechanical strain in CF is pronounced due to mucus plugging [34], heterogeneous air trapping [35], and chronic cough. The environment in the CF lung is therefore well primed for increased TGF-β signaling, myofibroblast differentiation and persistence of pathologic fibroblast behavior as established here.

In summary, our study describes a novel mechanism by which TGF-β associated myofibroblast differentiation contributes to the progression of cystic fibrosis lung disease. The development of experimental anti-fibrotic therapies, particularly those that limit TGF-β activation and signaling [36]–[43], could furnish a novel means to blunt the intense pulmonary fibrosis among CF subjects shown here. The findings provide a means by which TGF-β driven myofibroblast differentiation and dysfunction in CF lung disease can be better understood in the future.
