# Correction: Myofibroblast Differentiation and Enhanced Tgf-B Signaling in Cystic Fibrosis Lung Disease

**DOI:** 10.1371/annotation/d0132de3-56ca-4258-9119-bdab0ceb2cff

**Published:** 2013-08-29

**Authors:** William T. Harris, David R. Kelly, Yong Zhou, Dezhi Wang, Mark MacEwen, James S. Hagood, J. P. Clancy, Namasivayam Ambalavanan, Eric J. Sorscher

There was an error in the name of the fifth author.

The correct name is: Mark MacEwen

The correct citation is: Harris WT, Kelly DR, Zhou Y, Wang D, MacEwen M, et al. (2013) Myofibroblast Differentiation and Enhanced Tgf-B Signaling in Cystic Fibrosis Lung Disease. PLoS ONE 8(8): e70196. doi:10.1371/journal.pone.0070196 

